# Impacts of bariatric surgery in health outcomes and health care costs in Brazil: Interrupted time series analysis of multi-panel data

**DOI:** 10.1186/s12913-021-07432-x

**Published:** 2022-01-07

**Authors:** José Antonio Orellana Turri, Nana Kwame Anokye, Lionai Lima dos Santos, José Maria Soares Júnior, Edmund Chada Baracat, Marco Aurélio Santo, Flavia Mori Sarti

**Affiliations:** 1 Department of Gynecology and Obstetrics, Central Institute of the Hospital of Clinics at the School of Medicine, University of Sao Paulo, R Dr Eneas de Carvalho Aguiar 255, Sao Paulo, SP Brazil; 2 School of Public Health, University of Sao Paulo, Av Dr Arnaldo 715, Sao Paulo, SP Brazil; 3 Department of Clinical Sciences, College of Health and Life Sciences, Brunel University London, Kingston Lane, Uxbridge, United Kingdom; 4 Department of Physiotherapy, School of Sciences and Technology, Sao Paulo State University, Rua Roberto Simonsen, Presidente Prudente, SP 305 Brazil; 5 Department of Gastroenterology, Digestive Disease Surgery, Central Institute of the Hospital of Clinics at the School of Medicine, University of Sao Paulo, R Dr Eneas de Carvalho Aguiar 255, Sao Paulo, SP Brazil; 6 School of Arts, Sciences and Humanities, University of Sao Paulo, Av Arlindo Bettio 1000, Sao Paulo, SP Brazil

**Keywords:** Obesity, Bariatric surgery, Health care costs, Cohort, Health outcomes

## Abstract

**Background:**

The increasing burden of obesity generates significant socioeconomic impacts for individuals, populations, and national health systems worldwide. The literature on impacts and cost-effectiveness of obesity-related interventions for prevention and treatment of moderate to severe obesity indicate that bariatric surgery presents high costs associated with high effectiveness in improving health status referring to certain outcomes; however, there is a lack of robust evidence at an individual-level estimation of its impacts on multiple health outcomes related to obesity comorbidities.

**Methods:**

The study encompasses a single-centre retrospective longitudinal analysis of patient-level data using micro-costing technique to estimate direct health care costs with cost-effectiveness for multiple health outcomes pre-and post-bariatric surgery. Data from 114 patients who had bariatric surgery at the Hospital of Clinics of the University of Sao Paulo during 2018 were investigated through interrupted time-series analysis with generalised estimating equations and marginal effects, including information on patients' characteristics, lifestyle, anthropometric measures, hemodynamic measures, biochemical exams, and utilisation of health care resources during screening (180 days before) and follow-up (180 days after) of bariatric surgery.

**Results:**

The preliminary statistical analysis showed that health outcomes presented improvement, except cholesterol and VLDL, and overall direct health care costs increased after the intervention. However, interrupted time series analysis showed that the rise in health care costs is attributable to the high cost of bariatric surgery, followed by a statistically significant decrease in post-intervention health care costs. Changes in health outcomes were also statistically significant in general, except in cholesterol and LDL, leading to significant improvements in patients' health status after the intervention.

**Conclusions:**

Trends multiple health outcomes showed statistically significant improvements in patients' health status post-intervention compared to trends pre-intervention, resulting in reduced direct health care costs and the burden of obesity.

## Background

Obesity represents one of the major public health problems worldwide nowadays, generating significant socioeconomic impacts for individuals, populations, and national health systems. A higher prevalence of obesity has been associated with a higher occurrence of chronic non-communicable diseases, like type 2 diabetes *mellitus* (T2DM), hypertension, and cardiovascular diseases, leading to early mortality. The increase in Body Mass Index (BMI) that characterise obesity is associated with higher utilisation of health services and additional expenditures with medications, especially for the treatment of comorbidities [1–9].

Considering the rising costs of health care and the escalating burden of obesity in diverse countries, there have been numerous studies on the cost-effectiveness of health interventions towards preventing and treating obesity and its effects on comorbidities [10–15]. Recent evidence on obesity-related interventions' impacts encompasses the economic assessment of prevention [16–18] (promoting physical activity and healthy eating) and treatment (medication and/or surgical procedures) strategies [19–24].

Bariatric surgery is currently adopted as the standard treatment for moderate to severe obesity in diverse national health systems [19–24]. There are significant effects of bariatric surgery in decreasing body weight and improving health outcomes regarding cardiovascular events, T2DM, dyslipidemia, cancer, life expectancy, and quality of life [5, 23, 25–28] in the long run [29–32].

Direct costs of bariatric surgery usually range from US$25,000 to US$30,000 per patient. In contrast, the annual health care costs for patients with BMI≥35kg/m [2] generally vary between US$3,000 to US$10,000 for treatment of T2DM, hypertension, and other obesity-related conditions [3, 5, 33–35]. Obese individuals present approximately doubled risk for utilisation of medical services in comparison to eutrophic individuals (RR 1.89; CI95% 1.88-1.89, *p*<0.001), and mean annual medical costs are twofold higher in severe obesity (US$1,140 per capita) relative to the general population (US$567 per capita) [36]. Therefore, bariatric surgery may lead to cost savings estimated between US$1,209 to US$2,016 per patient due to reduction of adverse health outcomes and a decrease in the use of medication for the treatment of comorbidities [27, 37].

In Brazil, bariatric surgery was included in the list of procedures for treating moderate to severe obesity within the Brazilian Unified Health System (Sistema Unico de Saude, SUS) in 1999, therefore being accessible to eligible patients free of charge in public hospitals throughout the country [38, 39]. There have been increasing trends in the adoption of bariatric surgery in treating obesity, especially among young females [38], and results of previous studies have shown low mortality risk and high effectiveness of the surgery [24, 38, 40–44].

To date, studies focusing on the assessment of costs and effectiveness of bariatric surgery in Brazil and other countries have been based on limited sample size and/or single health outcome [5, 6, 23, 38] or relied on the modelling of future outcomes. There is limited evidence, including multiple anthropometric, hemodynamic, and biochemical parameters of patients [20, 22, 45], especially longitudinal data to compare health status and health care costs before and after bariatric surgery [27, 46].

Due to the high cost of bariatric surgery, decision-making processes regarding surgery adoption are limited by short term perspective focusing on direct costs in the absence of economic studies with long term outcomes. The significant increase in obesity rates in the country affects approximately 19% of the population in 2017 [47]. The lack of accurate information about costs and health outcomes involved in the surgery and pre-and post-hospitalisation periods delay the spread of the procedure throughout the Brazilian health system. Data on multiple health outcomes associated with health care costs from pre-bariatric surgery period until follow-up, at the patient level, may provide critical information for public policy decision making; particularly considering data from high complexity hospital considered reference health care institution in Brazil [48–53].

The present study addresses the literature gap by examining the long-term impact of bariatric surgery on direct health care costs and multiple health outcomes (including anthropometric, hemodynamic, and biochemical parameters), using an interrupted time-series approach (ITS) for analysis of patient-level data in Brazil. ITS's quasi-experimental design comprises a useful tool for evaluating longitudinal effects of interventions, mainly based on natural experiments occurring in real-world settings [54–60].

## Methods

### Study design

Quantitative analysis of a retrospective cohort of patients who had bariatric surgery at the Hospital of Clinics from the University of Sao Paulo (HC-FMUSP), Brazil, from January to December 2018, through Interrupted Time-Series Analysis (ITSA) on direct health care costs and health outcomes.

### Bariatric surgery characteristics

The patients included in the study sample were distributed in three groups of surgery: Roux-en-Y gastric by-pass (R-YGB), vertical gastrectomy, and adjustable gastric banding. In most cases, open surgery was performed; however, a minor proportion of patients had surgery through video laparoscopy.

According to the standard protocols for bariatric surgery within SUS, there is a requirement for previous assessment of patients' eligibility for bariatric surgery in primary health care facilities. Depending on the health status, patients are referred to a specialised health care unit. Furthermore, patients are required to perform numerous exams and consultations pre-and post-surgery on a regular basis in Brazil [39].

**Fig. 1:**
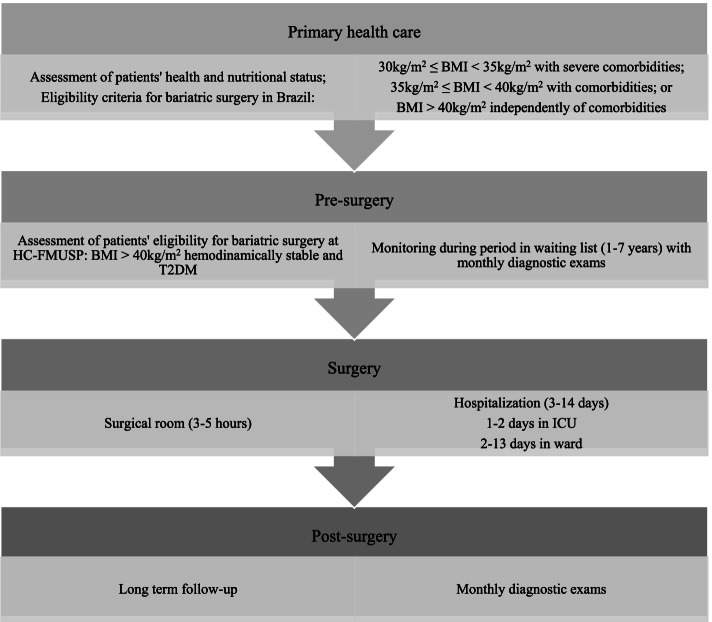
Flowchart of processes performed by patients eligible for bariatric surgery. Sao Paulo (Brazil), 2018. Obs.: BMI = Body mass Index; T2DM = type 2 diabetes mellitus; ICU = Intensive Care Unit

Patients with moderate to severe obesity diagnosis are referred to high complexity hospitals, like the HC-FMUSP, and included in the waiting list for bariatric surgery, performing monthly clinical and laboratory exams. After monitoring eligibility criteria during variable periods, patients are submitted to surgery, hospitalisation, and post-surgery follow-up, starting a post-surgical period of the monthly clinic and laboratory exams (Fig. 1).

### Sample

Data on a cohort of 121 patients who had bariatric surgery at the HC-FMUSP in Sao Paulo during 2018, Brazil, through the Brazilian Unified Health System (SUS) from January to December 2018, were obtained from the hospital's electronic medical records.

Only patients with complete data registered on the medical records were included in the study, encompassing information on multiple anthropometric, hemodynamic, and biochemical parameters through regular assessments of patients' health status, and utilisation of resources and costs of health care procedures, within six months pre-and post-intervention (bariatric surgery) [61]. Therefore, from 121 patients, data on 114 patients were included in the analysis.

The data was collected through the access of the electronic hospital records, using a filter for selecting patients with complete information in the fields encompassing the target variables of the study. The criteria for inclusion of patients in the study was established *a priori*: only patients with complete data during the period designated for the analysis would be selected to comprise the sample, that is, all patients who had bariatric surgery conducted during 2018 and complied with monitoring procedures established in the protocol during the six months pre-surgery and six months post-surgery were included in the dataset.

Individual information registered on daily-based electronic data collection at HC-FMUSP were gathered in a single dataset encompassing data on patients' characteristics, health outcomes (pre-and post-intervention), outpatient health care (pre-and post-intervention), and inpatient health care (pre-intervention, intervention, and post-intervention), including detailed information on utilisation of resources throughout the screening, intervention, and follow-up.

### Variables

The following patient information at baseline was obtained in medical records considering the periods of 180 days (6-months) pre-and post-intervention (Table 1).

**Table 1:** Information on health outcomes and utilisation of health care resources of patients from HC-FMUSP. Sao Paulo (Brazil), 2018

Variable	Components
Health outcomes	Anthropometric measures (weight and height); Hemodynamic measures (blood pressure); Biochemical exams (cholesterol and fractions, triglycerides, insulin, glucose-linked haemoglobin, and fasting glucose).
Health care costs	Outpatient health care: • Appointments with health professionals; • Clinical assessment; • Diagnostic exams. Inpatient health care referring to intervention (bariatric surgery) and obesity-related procedures pre-and post-intervention: • Hospital length of stay; • Type of surgery; • Inpatient procedures; • Use of resources including the operating room, medication, meals, human resources, hemodynamic and biochemical exams.

Health outcomes measures and outpatient health care costs were available monthly during pre-and post-surgery, and inpatient healthcare costs were available on a daily basis on the electronic hospital records.

The comparison of health outcomes and health care costs was based on the measurement of changes concerning the intervention's baseline (first day of hospitalisation for bariatric surgery).

Patients' demographic and lifestyle characteristics were also gathered to comprise control variables in statistical analysis, including gender and age, tobacco use, and alcohol consumption.

### Health outcomes

Information on health and nutritional status of patients, referring to the assessments pre-and post-surgery, were extracted in electronic medical records considering its associations with bariatric surgery in the literature [5, 23, 25–31], comprising the following set of health outcomes adopted for statistical analysis:

- Weight (kg);
- BMI (kg/m [2]);
- Blood pressure (mmHg);
- Cholesterol (mg/dL);
- VLDL (mg/dL);
- LDL (mg/dL);
- HDL (mg/dL);
- Triglycerides (mg/dL);
- Insulin (IU/mL);
- Glucose-linked haemoglobin (%);
- Fasting glucose (mg/dL)

Measurements of health outcomes were registered every month during screening, intervention, and follow-up by trained health professionals, according to standard procedures internationally recommended adopted within HC-FMUSP facilities.

### Health care costs

The study encompassed the measurement of direct health care costs pre-and post-bariatric surgery, i.e., costs directly related to the treatment of obesity and its comorbidities, including consultations, inpatient days, laboratory exams, image exams, and medication, using the perspective of the health care payer. The direct costs of obesity-related inpatient treatments pre- and post-intervention were reported separately from the direct costs of bariatric surgery in the study, considering that bariatric surgery also refers to treatment for obesity and simultaneously allows the analysis of trends pre-and post-surgery independently of bariatric surgery costs.

Data on utilisation of resources during outpatient and inpatient health care were used to estimate patient's direct health care costs referring to bariatric surgery, and 6-month pre-and post-intervention periods, adopting the health system perspective through the micro-costing approach.

The cost effectiveness was estimated in terms of cost per unit change in health outcomes, i.e., considering the total direct costs (in US$) required to change the health outcome in one unit (e.g., one kilogram for body weight, one mmHg for blood pressure, etc.).

Prices of inputs and wages of health professionals involved in health care procedures were obtained from the HC-FMUSP institutional database, based on information on inputs purchases and human resources payroll. Prices per item were multiplied by the amount used for the patient's treatment, and hourly wages were multiplied by the amount of time dedicated to performing procedures and consultations during the patient's treatment. Monetary values were updated to January 2020 and converted into US dollars using the Brazilian Central Bank official exchange rate.

### Statistical analysis

Descriptive statistics and interrupted time series analysis (ITSA) with generalised estimating equations (GEE) and marginal effects were performed using single-centre retrospective data on costs and multiple health outcomes related to bariatric surgery. The information gathered for the sample of patients from the HC-FMUSP was split into two segments for analysis, i.e., health outcomes and health care costs before and after bariatric surgery, respectively [61].

Dependent variables included in the models were health care costs and health outcomes (weight, BMI, blood pressure, cholesterol and fractions, triglycerides, insulin, glucose-linked haemoglobin, and fasting glucose).

GEE was fitted with uneven distribution for different outcomes during pre-intervention, intervention, and post-intervention to adjust monthly trends according to patients' characteristics. Marginal effects were obtained after GEE estimation by sample means at each evaluation period (pre-and post-intervention) and used to estimate incremental health care costs and effects for each health outcome.

Interrupted time series ordered logistic models were estimated for health outcomes, and Poisson models were estimated for health care costs, controlling for age and gender with random effects estimator. ITSA regression model uses a time series of a particular outcome of interest to establish an underlying trend interrupted by intervention at a given known point in time.

The model's statistical design draws an expected trend in the hypothetical scenario without the intervention, compared with the new trend established post-intervention to identify potential differences throughout time. The post-intervention scenario provides a comparison for the evaluation of the intervention impacts by calculating the change in slope throughout time, according to the following standard equation: [62, 63]

1 $${Y}_t={\beta}_0+{\beta}_1{\mathrm{T}}_t+{\beta}_2{X}_t+{\beta}_3{X}_t{\mathrm{T}}_t+{\varepsilon}_t$$

Y_t_ is the accumulated result measured at each spaced time point t, *Τ*_t_ is the time since the start of the study, X_t_ is a dummy (indicator) variable representing the intervention (pre-intervention periods 0, or 1) *X*_*t*_ *Τ*_*t*_ is an interaction term [63]. The regression coefficient for *T*_*t*_ represents the rate of change of activity in stage 1, and the sum of regression coefficients for *T*_*t*_ and (*XT*)_*t*_ is the rate of change of activity in stage 2. The effect over time was defined as the difference in the rate of change from stage 1 to stage 2, that is, the regression coefficient of (*XT*)_*t*_. β0 symbolises the starting level of the outcome variable. β1 is the slope or trajectory of the outcome variable until the introduction of the intervention. β2 represents the variation in the level of the variable that take place in the period immediately following the introduction of the intervention (the bariatric surgery). β3 represents the difference between pre-intervention and post-intervention slopes of the outcome. Thus, significant p-values in the post-intervention period to indicate the treatment effect of the bariatric surgery over time.

Thus, interrupted time series models were achieved by defining independent variables *Δt* = time point (1, 2, 3, 4, 5, 6, 7, 8, 9, 10, 11, 12, 13), *X*_*t*_ =0 for time points in stage 1 (time points 1, 2, 3, 4, 5 and 6) and 1 for time points in stage 2 (time points 7, 8, 9, 10, 11, 12 and 13), and (*XT*)_*t*_ =0 for time points in stage 1, and (t−7) for time points in stage 2.

The intervention's immediate effect was defined as the regression coefficient corresponding to *X*_*t*_ (corresponding to the counterfactual difference between stage 1 and stage 2 evaluated at time point 7). The interrupted series time models for health costs and outcomes were ordinal logistic repeated measures models, including additive effects for *T*_*t*_, *X*_*t*_ and (*XT*)_*t*_, and adjustment covariates.

The post-trend was calculated through ITSA estimation, considering the measures in the first period (pre-intervention) as baseline parameter for comparison concerning the measures of the second period (post-intervention). ITSA approach presents results similar to generalised non-linear regression models; however, instead of only one regression, ITSA considers the interruption at one specific point within the model, tracing two regressions for the variable and indicating statistical differences between trends pre-and post- interruption of the time series.

The statistical analysis was conducted using Stata version 14, and Newey-West standard errors were reported to account for autocorrelation at lag 1 [59].

## Results

### Characteristics of patients at baseline, intervention and follow-up

Considering characteristics at baseline, most patients were female, approximately 48 years old. Most patients had comorbidities, especially hypertension and/or diabetes, and a small proportion of individuals were smokers. The type of surgery conducted in most cases was open Y-Roux gastric bypass (Table 2).

**Table 2:** Baseline characteristics of bariatric surgery patients. Sao Paulo (Brazil), 2018

Gender		N	(%)
Men		9	7.9
Women		95	83.3
**Age (μ±SD)**		**47.8 ± 14.1**	
**Lifestyle characteristics**			
Tobacco use		20	17.5
Alcohol consumption		0	0.0
**Comorbidity**		**N**	**(%)**
Hypertension		72	63.2
T2DM		67	58.8
Sleep apnea		31	27.2
Dyslipidemia		25	21.9
Arthrosis		25	21.9
Hypothyroidism		10	8.8
Varices		10	8.8
CRI		10	8.8
CCI		5	4.4
**Type of surgery**		**N**	**(%)**
Y-Roux gastric by-pass	Open	67	58.8
	Video laparoscopic	34	29.8
Sleeve (vertical gastrectomy)	Open	4	3.5
	Video laparoscopic	3	2.6
Adjustable gastric banding	Open	3	2.6
	Video laparoscopic	3	2.6

Obs.: *μ* mean, *SD* standard deviation, *T2DM* type 2 diabetes mellitus, *CRI* chronic renal insufficiency, *CCI* Cardiac Congestive Insufficiency

Health outcomes presented improvement after intervention in general, except cholesterol and VLDL. There was an increase in HDL and decreased weight, BMI, LDL, triglycerides, insulin, glucose-linked haemoglobin, and glucose, showing improvements in patients' health status after bariatric surgery (Table 3).

**Table 3:** Health outcomes and direct health care costs of patients during 6-month pre-and post-bariatric surgery. Mean values of each period. Sao Paulo (Brazil), 2018

**Health outcomes**	**Pre-surgery μ (±SD)**	**Post-surgery μ (±SD)**	***P***	**Cost per unit change (US$)**
Weight (kg)	125.53 (±18.58)	103.23 (±24.46)	**<0.001**	61.68
BMI (kg/m [2])	47.36 (±4.22)	39.01 (±7.26)	**<0.001**	164.71
Blood pressure (mmHg)	132 (±15.21)	126.51 (±17.36)	**0.0025**	250.52
Cholesterol (mg/dL)	165.11 (±39.9)	167.78 (±38.55)	0.8341	515.12
VLDL (mg/dL)	21.51 (±8.33)	22.91 (±8.93)	**<0.001**	982.41
LDL (mg/dL)	94.06 (±34.66)	93.2 (±34.58)	**0.0147**	1,599.27
HDL (mg/dL)	45.63 (±14.38)	51.88 (±13.23)	**<0.001**	220.06
Triglycerides (mg/dL)	130.67 (±64.21)	117.27 (±56.95)	**<0.001**	102.64
Insulin (IU/mL)	17.19 (±22.55)	12.69 (±7.47)	**<0.001**	305.64
Glucose-linked hemoglobin (%)	127.57 (±43.88)	113.61 (±36.47)	**0.001**	98.52
Fasting glucose (mg/dL)	113.22 (±45.77)	99.11 (±39.83)	**<0.001**	97.47
**Health care costs**	**Pre-surgery μ (±SD)**	**Post-surgery μ (±SD)**	***P***	**Cost per unit change (US$)**
Consultations	17.64 (±13.9)	18.06 (±15.32)	**<0.001**	3,274.69
Inpatient days*	11,913.26 (±12,682.01)	9,151.04 (±9,695.16)	**<0.001**	0.50
Laboratory exams	108.72 (±112.94)	177.68 (±217.78)	**<0.001**	19.94
Image exams	888.99 (±901.22)	881.46 (±854.78)	**0.0161**	182.65
Medication	683.9 (±854.73)	508.45 (±696.99)	**<0.001**	7.84
Total direct costs	996.61 (±4,515.39)	2,371.98 (±6,172.79)	**<0.001**	-

Obs.: *μ* mean, *SD* standard deviation. *Including the cost of bariatric surgery

Mean direct costs of hospitalization (-US$2,762.22; -23.2%), image exams (-US$7.53; -0.8%) and medication (-US$175.37; -25,7%) presented decrease after bariatric surgery. On the other hand, total direct costs (US$1,375.37; +138%), consultations (US$0.42; +2.4%) and laboratory exams (US$68.96; +63.4%) increased after intervention, especially due to need of patients’ follow-up after surgery. The direct cost was per US$ 61.68 kilogram of body weight, and US$ 164.71 per unit of BMI decreased per patient (Table 3).

### Changes between pre-and post-intervention using ITSA

The interrupted time series analysis showed a rise in overall health care costs at the intervention period due to the high cost of bariatric surgery; however, it was followed by a statistically significant decrease in post-intervention health care costs. Post-surgery changes in health outcomes were also statistically significant in general, except in cholesterol and LDL, leading to significant improvements in patients' health status after the intervention.

The differences in health outcomes and direct costs between pre-surgery and post-surgery periods are presented in Table 4. The value of the constant refers to the baseline measurement, six months before the bariatric surgery, whilst pre-intervention values represent the point values registered in the first month before the surgery, intervention values represent the first measurement post-surgery, and post-intervention values represent the point values registered in the first month after the surgery. Estimates considered the reference period based on the date of each patient’s first intervention, and calculated the level of increase or decrease in values in the following period.

**Table 4:** Pre-, intervention and post-intervention scenario of bariatric surgery using ITSA (point values referring to the first date of data acquisition). Sao Paulo (Brazil), 2018

Health outcomes	Cons	Pre			Int			Post		
		Coef	95% CI	P*	Coef	95% CI	P*	Coef	95% CI	P*
**Weight**	133.7986	0.0053	(-.0029; 0.0134)	0.208	-15.7546	(-20.9373; -10.5312)	**<0.001**	-0.0324	(-0.0441; -0.0205)	**<0.001**
**BMI**	53.6564	-0.0024	(-0.0025; 0.0029)	0.894	4.4470	(2.0075; 7.4842)	**0.001**	-0.0158	(-0.0256; -0.0162)	**<0.001**
**Blood pressure**	132.7641	-0.0024	(-0.0081; 0.0032)	0.402	0.4449	(-3.2024; 4.0685)	0.815	-0.0061	(-0.0140; 0.0018)	0.131
**Cholesterol**	157.4912	-0.0170	(-0.0331; -0.0008)	**0.039**	7.1876	(-0.8418; 15.1757)	0.079	0.0186	(-0.0013; 0.0385)	0.066
**VLDL**	19.3720	0.0043	(0.0014; 0.0072)	**0.004**	3.1873	(1.3006; 5.0683)	**0.001**	-0.0124	(-0.0164; -0.0085)	**<0.001**
**LDL**	93.4065	-0.0093	(-0.0233; 0.0047)	0.194	9.2046	(1.8887; 16.5348)	**0.014**	-0.0080	(-0.0257; 0.0096)	0.373
**HDL**	33.0861	0.0212	(0.0156; 0.0268)	**<0.001**	-8.5178	(-11.6697; -5.3983)	**<0.001**	-0.0024	(-0.0098; 0.0049)	0.523
**Triglycerides**	108.6332	0.0231	(-0.0066; 0.0528)	0.128	0.4112	(-17.1356; 17.8948)	0.966	-0.0775	(-0.1176; -0.0375)	**<0.001**
**Insulin**	9.9325	0.0150	(0.0108; 0.0193)	**<0.001**	-5.2224	(-7.4075; -3.0374)	**<0.001**	-0.0209	(-0.0263; -0.0156)	**<0.001**
**Glucose-linked haemoglobin**	126.1915	0.0038	(-0.0156; 0.0231)	0.703	-3.6614	(-14.8040; 7.4856)	0.520	-0.0326	(-0.0580; -0.0071)	**0.012**
**Fasting glucose**	101.4913	0.0343	(0.0143; 0.0545)	**0.001**	-12.6732	(-23.9196; -1.4268)	**0.027**	-0.0719	(-0.0954; -0.0485)	**<0.001**
**Direct costs**	180.27	0.8673	(0.0750; 1.6596)	**0.032**	4,700.29	(3,452.16; 5,948.43)	**<0.001**	-10.2397	(-12.5172; -7.9624)	**<0.001**

Obs.: *cons* constant, *pre* pre-intervention, *int* intervention, *post* post-intervention. Regression with Newey-West standard errors. *Statistical significance of coefficients

### Post-intervention linear trends

**Fig. 2:**
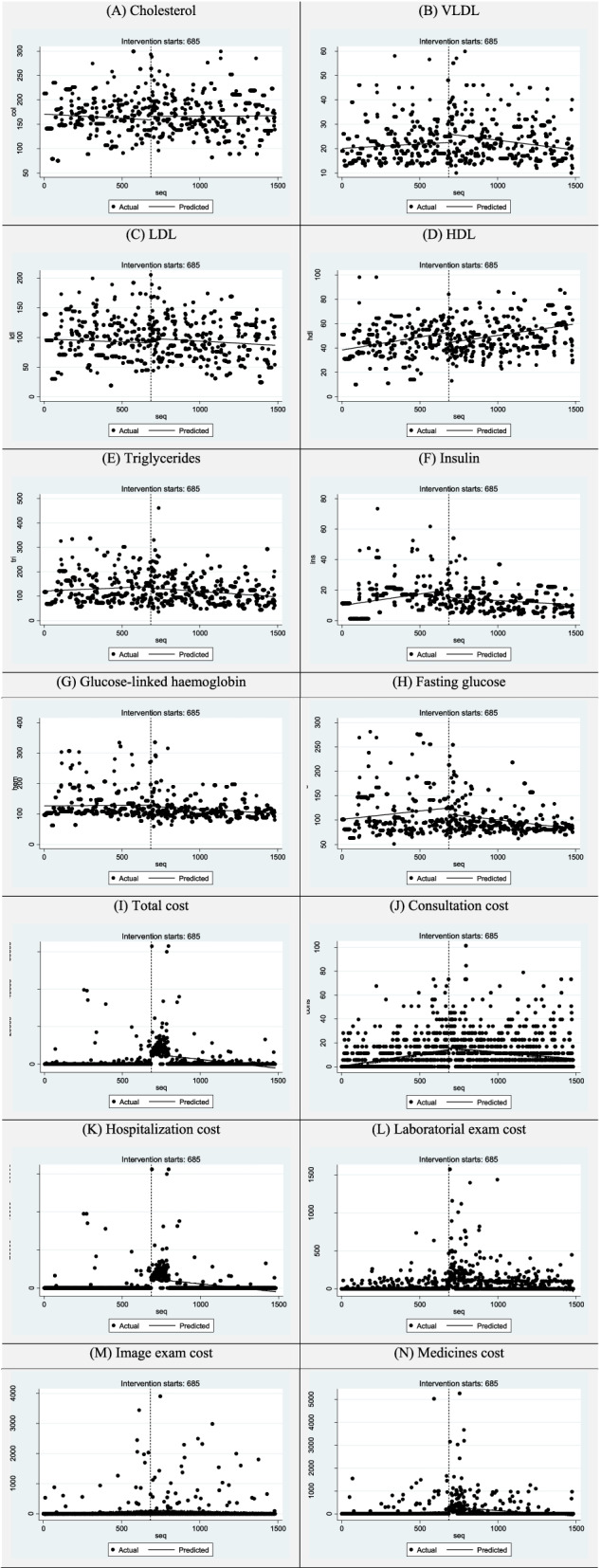
Trends of direct costs and health outcomes of bariatric surgery using ITSA for 180-day period pre-and post-intervention. Sao Paulo (Brazil), 2018

Trends in post-intervention health outcomes showed improvement in patients' health status, except for cholesterol. For each measurement, the post-intervention trend shows the monthly trend to a descendant or ascendant value or US$ (for health-related expenditure) after the bariatric surgery. However, the positive trend post-intervention in cholesterol was not statistically significant. BMI, VLDL, HDL, and fasting glucose showed significant changes in trends pre-and post-surgery (Table 5 and Fig. 2).

**Table 5:** Post-intervention trends of health outcomes of bariatric surgery. Sao Paulo (Brazil), 2018

Health outcomes	Post intervention trend	CI 95%	***p***
Weight	-0.0271	(-0.0356;-0.0186)	**<0.001**
BMI	-0.0208	(-0.0246;-0.0169)	**<0.001**
Blood pressure	-0.0085	(-0.0141;-0.0030)	**0.0025**
Cholesterol	0.0016	(-0.0100;0.0133)	0.7820
VLDL	-0.0082	(-0.0108;-0.0055)	**<0.001**
LDL	-0.0173	(-0.0281;-0.0066)	**0.0016**
HDL	0.0188	(0.0150;0.0226)	**<0.001**
Triglycerides	-0.0544	(-0.0755;-0.0334)	**<0.001**
Insulin	-0.0058	(-0.0083;-0.0034)	**<0.001**
Glucose-linked hemoglobin	-0.0288	(-0.0414;-0.0162)	**0.001**
Fasting glucose	-0.0376	(-0.0470;-0.0281)	**<0.001**
Total direct cost	-9.3725	(-11.5077;-7.2373)	**<0.001**
Consultations	-0.0113	(-0.0170;-0.0056)	**0.001**
Hospitalizations	-8.5584	(-10.6245;-6.4924)	**<0.001**
Laboratory exams	-0.2202	(-0.2797;-0.1608)	**<0.001**
Image exams	-0.1059	(-0.1923;-0.0194)	**0.0164**
Medication	-0.4767	(-0.6364;-0.3170)	**<0.001**

Obs.: CI 95% = 95% confidence interval. Regression with Newey-West standard errors

The generalised estimating multivariate regression controlling for patients' characteristics showed that changes observed in the comparison between pre-and post-surgery remain statistically significant. Marginal effects in direct health care costs post-intervention, including covariates, were significantly negative, similarly to health outcomes referring to blood pressure, VLDL, triglycerides, insulin, glucose-linked haemoglobin, and fasting glucose (Table 6).

**Table 6:** Marginal effects during pre-intervention, intervention, post-intervention adjusted by gender and age using GEE. Sao Paulo (Brazil), 2018

**Health Outcomes**	**Characteristics**	**Dy/dx**	**95%CI**	***P***
BMI	Pre	0.003	(0.001; 0.005)	**<0.001**
	Int	0.803	(0.006; 0.154)	**0.033**
	Post	-0.0006	(-0.0008; -0.0004)	**<0.001**
	Gender (female)	-0.005	(-0.034; 0.024)	0.744
	Age	0.0007	(-2.855; 3.001)	0.105
Weight	Pre	0.002	(-0.01; -0.01)	0.804
	Int	-58.404	(-69.9; -46.9)	**<0.001**
	Post	0.086	(0.06; -0.1)	**<0.001**
	Gender (female)	-4.286	(-5.29; -3.27)	**<0.001**
	Age	-0.12	(-0.15; -0.08)	**<0.001**
Blood pressure	Pre	-0.002	(-0.005; 0.001)	0.167
	Int	0.522	(-1.66; 2.71)	0.640
	Post	-0.006	(-0.01; -0.001)	**0.007**
	Gender (female)	0.221	(-1.6; 2.04)	0.812
	Age	0.007	(-0.04; 0.06)	0.808
Cholesterol	Pre	-0.03	(-0.04; -0.01)	**<0.001**
	Int	6.047	(-0.78; 12.87)	0.083
	Post	0.046	(0.03; 0.06)	**<0.001**
	Gender (female)	9.214	(7.43; 10.98)	**<0.001**
	Age	0.121	(0.06; 0.17)	**<0.001**
VLDL	Pre	0.007	(0.003; 0.01)	**<0.001**
	Int	1.784	(0.14; 3.42)	**0.033**
	Post	-0.014	(-0.01; -0.01)	**<0.001**
	Gender (female)	-0.108	(-0.75; 0.54)	0.744
	Age	0.016	(-0.003; 0.03)	0.105
LDL	Pre	-0.017	(-0.02; -0.006)	**0.002**
	Int	-1.776	(-7.95; 4.39)	0.573
	Post	0.033	(0.01; 0.04)	**<0.001**
	Gender (female)	6.900	(5.58; 8.21)	**<0.001**
	Age	-0.039	(-0.07; -0.0001)	**0.049**
HDL	Pre	0.022	(0.01; 0.02)	**<0.001**
	Int	-8.427	(-10.16; -6.69)	**<0.001**
	Post	-0.005	(-0.008; -0.0006)	**0.023**
	Gender (female)	1.125	(0.14; 2.1)	**0.024**
	Age	0.092	(0.06; 0.12)	**<0.001**
Triglycerides	Pre	0.051	(0.04; 0.06)	**<0.001**
	Int	-6.802	(-11.98; -1.61)	**0.01**
	Post	-0.111	(-0.12; -0.09)	**<0.001**
	Gender (female)	6.805	(5.27; 8.33)	**<0.001**
	Age	0.18	(0.13; 0.22)	**<0.001**
Insulin	Pre	0.014	(0.01; 0.01)	**<0.001**
	Int	-7.207	(-9.05; -5.35)	**<0.001**
	Post	-0.017	(-0.02; 0.01)	**<0.001**
	Gender (female)	0.207	(-0.3; 0.71)	0.427
	Age	-0.001	(-0.01; 0.01)	0.885
Glucose-linked haemoglobin	Pre	0.013	(0.003; 0.02)	**0.007**
	Int	-3.227	(-8.41; 1.96)	0.223
	Post	-0.051	(-0.06; 0.03)	**<0.001**
	Gender (female)	4.468	(2.96; 5.97)	**<0.001**
	Age	-0.011	(-0.05; 0.03)	0.643
Fasting glucose	Pre	0.019	(0.006; 0.03)	**0.003**
	Int	8.873	(1.76; 15.98)	**0.014**
	Post	-0.095	(-0.11; -0.07)	**<0.001**
	Gender (female)	6.721	(5.37; 8.06)	**<0.001**
	Age	0.119	(0.07; 0.15)	**<0.001**
**Health Care Costs**	**Characteristics**	**Dy/dx**	**95%CI**	***P***
Direct cost	Pre	1.153	(1.12; 1.17)	**<0.001**
	Int	1189.518	(1179.29; 1199.74)	**<0.001**
	Post	-4.323	(-4.35; -4.29)	**<0.001**
	Gender (female)	-100.838	(-103.32; -98.34)	**<0.001**
	Age	1.303	(1.22; 1.38)	**<0.001**

Obs.: *pre* pre-intervention, *int* intervention, *post* post-intervention

## Discussion

Obesity imposes significant socioeconomic and health burden on individuals, health systems, and societies worldwide; therefore, its prevention and treatment may represent substantial impacts on health status, quality of life, and healthcare resources utilisation. Results presented in the study showed a reduction in direct health care costs and improvements in multiple health outcomes in a cohort of patients who had bariatric surgery in a reference hospital within the Brazilian health system, comparing 180-day period pre-and post-intervention through interrupted time series analysis with generalised estimating equations, controlling for individual characteristics.

The adoption of the micro-costing technique allowed to identify the leading health care cost drivers pre-and post-surgery in a high complexity institution from the public sector in Brazil. Although there was an initial rise in overall health care costs during bariatric surgery, the post-intervention trends were significantly negative, showing the potential for reducing obesity-related health care costs within the national health system.

Pre-intervention results allowed the analysis of cumulative health care costs related to procedures related to comorbidities, screening and monitoring during the waiting list for bariatric surgery. Information on health outcomes showed trends in worsening of patients' health status due to obesity-related comorbidities during the period before the intervention, mainly referring to weight gain, hypertension, T2DM, and dyslipidemia [23, 64].

On the other hand, post-intervention trends in health outcomes showed general improvement of patients' health status, especially indicators related to hypertension, T2DM, and dyslipidemia (except cholesterol, without statistical significance), controlling for patients' characteristics. A previous study on the health impacts of bariatric surgery in Brazil showed a reduction in the prevalence of T2DM, hypertension and dyslipidemia after 36 months of the surgery, estimating a reduction in medication and health care costs during the post-operative period [41]. However, the study failed to include costs of the bariatric surgery or its complications, therefore underestimating the overall costs involved in the intervention [41].

Evidence from other previous studies indicate that bariatric surgery resulted in improvement of patients' general health conditions and quality of life, including control and/or reduction of adverse health outcomes from obesity-related comorbidities, like hypertension and diabetes, generating relatively low costs to the public health system [3, 15, 65].

The changes in health outcomes resulted in negative marginal effects in post-intervention direct health care costs, similarly to results from a systematic review with meta-analysis recently published [64]. In addition to approximately 50% reduction in costs of medication for obesity-related comorbidities, there was a 78% decline in the prevalence of hypertension and 92% decrease in the prevalence of T2DM post-intervention, associated with a reduction in the use of anti-hyperglycemic medication in 93% of diabetic patients after surgery, and decrease in the adoption of anti-hypertensive medication in 48% of hypertensive patients [64].

Cost-effectiveness ratios estimated in the study showed reasonable costs per unit of health outcome obtained in the context of the national health system. The estimated direct cost of US$61.68 per kg of weight loss and approximately US$100.00 per unit of decrease in T2DM biomarkers, like glucose-linked haemoglobin and fasting glucose, showed that bariatric surgery presented costs similar to drug therapy in the country: the annual expenditure for T2DM treatment with medication is approximately US$260.00 per patient in Brazil, whilst in the rest of the world it ranges from US$1,937 to US$13,243 (or US$63,722 during a 35-year lifetime) [6, 33, 66–68].

Regarding biochemical markers for dyslipidemia, which are positively associated with cardiovascular risk and early mortality, the results showed cost-effectiveness ratios of US$102.64 and US$ 982.41 per unit for a reduction of triglycerides and VLDL, respectively. The average annual cost in medicines for the treatment of dyslipidemia varies between US$1,417 and US$2,300 [41]. Therefore, cost-effectiveness ratios of bariatric surgery regarding reduction in triglycerides and VLDL may be considered low cost compared to costs for treatment of dyslipidemia, especially considering benefits of the long-term prevention of cardiovascular events [69, 70].

To date, traditional observational studies assessed the costs involved in procedures and/or measurement of bariatric surgery's effectiveness using average pre-and post-intervention data for comparison of limited health outcomes. However, it is an approach that neglects to identify individual-level trends in patients' costs and health outcomes during pre-and post-surgery, especially considering that various studies fail to account for a comprehensive set of health care costs [41, 42, 68, 71, 72].

The present study provides additional evidence regarding the impacts of bariatric surgery on direct health care costs and multiple health outcomes related to comorbidities of moderate to severe obesity with an in-depth analysis of trends using individual-level data. The combination of ITS and GEE modelling approaches allowed us to assess the marginal effects of bariatric surgery in the evolution of costs and outcomes, including correction for patients' characteristics, reducing the potential bias of estimation.

The results suggest the existence of a causal relationship between bariatric surgery and improvement of health outcomes in patients with severe to moderate obesity, considering that patients were followed during similar periods before (waiting list) and after (follow-up) bariatric surgery to provide balanced information pre-and post-intervention for comparison of health care costs and health outcomes. The robustness of results indicates efficiency and effectiveness of bariatric surgery in interruption of patients' worsening health conditions and promotion of improvements in health outcomes related to obesity-related comorbidities like diabetes, hypertension, and dyslipidemia; thus resulting in a reduction of direct health care costs after treatment, findings in accordance with previous evidence [15, 19–21, 23, 24].

Due to the lack of previous evidence regarding impacts and cost-effectiveness of bariatric surgery with multiple health outcomes using the micro-costing approach, the estimates obtained may provide real-world foundations for public policy decision making in national health systems, especially referring to intervention with significant potential for reduction in the burden of disease related to obesity [71, 72].

The study's main limitation refers to the sample size of patients with moderate to severe obesity within one Brazilian hospital; however, it is essential to highlight the extensive criteria for patients' inclusion in the sample to develop robust statistical analysis. The selection of patients to comprise the sample for interrupted time series analysis with GEE was based on strict eligibility of patients considering the existence of complete information on multiple health outcomes registered in electronic medical records encompassing periods pre-and post-intervention and the requirement of detailed data on utilisation of resources within the health system to perform micro-costing approach for estimation of costs. Further investigation on the cost-effectiveness of bariatric surgery should be performed using patient-level longitudinal data, encompassing sample with representativeness at the population level, to allow further robustness in assessing trends regarding the intervention's health care costs and health outcomes.

## Conclusions

Bariatric surgery represents an efficient and effective intervention for treating moderate to severe obesity, encompassing extensive benefits in health outcomes and supporting other public health strategies towards health promotion and reducing disease burden. Trends in multiple health outcomes showed statistically significant improvements in patients' health status post-intervention compared to trends pre-intervention, resulting in reduced direct health care costs and burden of obesity, leading to a decrease in the risk of mortality potential increase in quality of life.

## Data Availability

The datasets generated and/or analysed during the current study are not publicly available due to the necessity of the Hospital of Clinics of the University of Sao Paulo (Brazil) permission. However, data are available from the corresponding author on reasonable request.
