# Correction to: Impacts of bariatric surgery in health outcomes and health care costs in Brazil: Interrupted time series analysis of multi-panel data

**DOI:** 10.1186/s12913-022-07486-5

**Published:** 2022-01-17

**Authors:** José Antonio Orellana Turri, Nana Kwame Anokye, Lionai Lima dos Santos, José Maria Soares Júnior, Edmund Chada Baracat, Marco Aurélio Santo, Flavia Mori Sarti

**Affiliations:** 1 Department of Gynecology and Obstetrics, Central Institute of the Hospital of Clinics at the School of Medicine, University of Sao Paulo, R Dr Eneas de Carvalho Aguiar 255, Sao Paulo, SP Brazil; 2 School of Public Health, University of Sao Paulo, Av Dr Arnaldo 715, Sao Paulo, SP Brazil; 3 Department of Clinical Sciences, College of Health and Life Sciences, Brunel University London, Kingston Lane, Uxbridge, United Kingdom; 4 Department of Physiotherapy, School of Sciences and Technology, Sao Paulo State University, Rua Roberto Simonsen, Presidente Prudente, SP 305 Brazil; 5 Department of Gastroenterology, Digestive Disease Surgery, Central Institute of the Hospital of Clinics at the School of Medicine, University of Sao Paulo, R Dr Eneas de Carvalho Aguiar 255, Sao Paulo, SP Brazil; 6 School of Arts, Sciences and Humanities, University of Sao Paulo, Av Arlindo Bettio 1000, Sao Paulo, SP Brazil

**Correction to: BMC Health Serv Res 22, 41 (2022)**

**https://doi.org/10.1186/s12913-021-07432-x**

Following publication of this article [1], the corresponding author should be changed from José Antonio Orellana Turri to Nana Kwame Anokye due to a typesetting error.

The original article [1] has been corrected.
